# The effect of activities of daily living on anxiety in older adult people: the mediating role of social participation

**DOI:** 10.3389/fpubh.2024.1450826

**Published:** 2024-12-13

**Authors:** Juan Zheng, Jianqiang Xu, Daqi Liu

**Affiliations:** School of Management, Xuzhou Medical University, Xuzhou, Jiangsu, China

**Keywords:** older adults, activities of daily living, social participation, anxiety, mediating effect

## Abstract

**Objective:**

Anxiety is a serious psychiatric illness in older adult people. Activities of daily living and social participation are important factors influencing anxiety in older adult people. Using social participation as a mediating variable, this study explored the influence of activities of daily living on anxiety in older adult people to provide scientific evidence for improving the psychological health of older adult people.

**Methods:**

A multistage stratified random sampling method was used in this study to recruit older adult people. T tests and variance analysis were used for single factor analysis, and a multiple linear regression model was used for multifactor analysis. Pearson correlation analysis was used to study the correlation between activities of daily living and social participation and anxiety. The Process program was used to perform the bootstrap test.

**Results:**

The mean anxiety score of the interviewed older adult individuals was 3.028 ± 4.606 points, and 28.66% of the older adult individuals had anxiety. There was a statistically significant difference in the anxiety scores of older adult people across sex, age, marital status, education level, and health insurance status (*p* < 0.05). After controlling for other variables, each one-point increase in the activities of daily living score significantly increased the anxiety score by 0.122 points (𝛽=0.122, *p* < 0.001), and each one-point increase in the social participation score significantly decreased the anxiety score by 0.058 points (𝛽= − 0.058, *p* < 0.001). According to the results of the mediating effect analysis, there was a significant positive relationship between activities of daily living and anxiety, and the total effect was established (𝛽=0.1719, *p* < 0.001). After controlling for social participation, there was a significant direct effect of activities of daily living on anxiety in older adult people (𝛽=0.1255, *p* < 0.001). A partial mediating effect of social participation on the relationship between activities of daily living and anxiety in older adult people was found. The indirect effect of social participation was 26.99%.

**Conclusion:**

Activities of daily living and social participation are important factors affecting anxiety in older adult people. Health education should be increased to enhance the physical activity of older adult people to improve their activities of daily living, create a good atmosphere for their social participation, improve their motivation for and continuity of social participation.

## 1 Introduction

With the development of society, aging has gradually become the symbol and product of modern society. In 2000, China became an aging society. On the one hand, the absolute size of the older adult population has increased; on the other hand, the relative size of the older adult population has been increasing. According to the seventh demographic census, China’s older adult population reached 260 million, accounting for 18.7% of the total population (1). With the increasing number of older adult people, the physical and psychological health of older adult people has become increasingly important to society. Anxiety is a serious psychiatric illness among these individuals (2). According to the World Health Organization (WHO), 3.8% of older adult people in the world experience anxiety (3). Previous literature found that the prevalence of anxiety and anxiety symptoms in older adults ranged from 1.2 to 15% and 15 to 52.3%, respectively (4, 5). As one of the most common diseases in later life, anxiety cannot be found and treated timely (6). Research has shown that anxiety disorders, common mental disorders in people aged 65 years or older (7), not only reduce quality of life (8) but also increase the risk of death and other diseases (9). Anxiety and depression are closely related, and both can cause serious harm to physical and psychological health (10, 11). Active interventions to prevent the onset and exacerbation of anxiety in older adult people can effectively reduce the risk of heart disease and chronic noncommunicable respiratory diseases (12).

As a common psychological problem, anxiety is affecting an increasing number of older adult people, but its diagnosis and treatment status have not reached the ideal level in all countries worldwide. More than 25% of Americans have experienced anxiety, but most of them did not receive treatment (13). A Norwegian study of 65,648 respondents revealed that 75% of the respondents with anxiety never sought help (14). Instead, they let it progress. China’s medical resources are relatively scarce, and the levels of diagnosis and treatment of anxiety are low. The hospitalization of older adult people, who are a vulnerable group in society, is not optimal. The treatment of anxiety and depression in older adult people is difficult, and the effect is not ideal. Therefore, it is particularly important to clarify the factors influencing anxiety and prevent anxiety in older adult people. According to the statistics of the WHO and a survey of researchers, social and economic levels are prominent factors affecting anxiety, followed by race, ethnicity, gender, age, living habits, regional differences, etc. (15, 16). In addition, previous studies have confirmed that physical diseases are correlated with the incidence of anxiety (17–21). Physical health is an important factor influencing anxiety in older adult people.

Activities of daily living (ADL) refers to physical movements that people must repeat every day to live independently (22). ADL is an important indicator of the physical health of older adult people and is also important factor influencing anxiety (23). A decrease in self-care ability tends to cause a psychological burden and lead to adverse emotional experiences, seriously affecting the psychological health of older adult people (24). Studies have shown that the impairment of activities of daily living may affect the psychological health of older adult people, especially their anxiety and depression. Some studies have even concluded that the impairment of activities of daily living is the only independent risk factor for anxiety and depression (25). It can be seen that the ability of daily living is closely related to psychological problems. Impaired ADL reduces the range of activities of the older adult, reduces the opportunity for the older adult to contact with the outside world and exchange information, and makes them prone to psychological states such as loneliness, anxiety and irritability, which can lead to mental problems. Meanwhile, the impairment of activities of daily living in older adult people, to varying degrees, leads to a significant decrease in social participation and seriously affects their quality of life (26).

**Figure 1:**
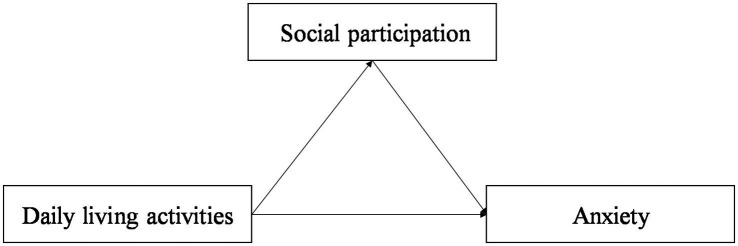
The hypothesis model.

Social participation is the core component of active aging and a necessary way to achieve active aging. Social participation is also an important factor influencing the psychological health of older adult people and an important way to promote psychological health and improve quality of life in older adult people. The research shows that social participation has a significant impact on the psychological health of older adult people. With increasing social participation, the psychological state of older adult people tends to improve (27). Nancy’s study revealed that older adult people’s participation in volunteer services not only expands their social circles but also helps them realize their own value and improve their quality of life (28). It can be seen that social participation helps to improve the psychological health level and quality of life and reduce the occurrence of psychological problems such as anxiety of the older adult person. In summary, activities of daily living and social participation have a certain impact on the anxiety of the older adult. Activities of daily living affect also significantly affects social participation. However, there is little research on the relationship between ADL, social participation, and anxiety. Therefore, we hypothesized that the anxiety is the dependent variable, ADL is the independent variable, and social participation is the mediating variable, so as to explore the influencing mechanisms of the effects of activities of daily living on anxiety in older adult people, so as to provide a scientific basis for reducing anxiety, improving psychological health and improving quality of life in the older adult people. Therefore, we propose the following hypothesis: (1) ADL has a significant effect on the social participation and anxiety of the older adult; (2) Social participation has a significant effect on the anxiety of the older adult; (3) Social participation plays a mediating role between ADL and anxiety. Figure 1 illustrates the hypothesis model of the study.

## 2 Materials and methods

### 2.1 Study design and respondents

This study included a survey on the health and aging of older adult people in Jiangsu Province, China. A multistage stratified random sampling method was used. Three prefectural-level cities representing different levels of economic development and aging in Jiangsu Province were first selected, and then older adult people were randomly selected from two urban and two rural communities in each prefectural-level city. The inclusion criteria for the survey respondents were (1) aged ≥60 years; (2) long-term residence at the survey site for more than 6 months; (3) normal language expression and good communication skills; and (3) voluntary completion of the survey. The exclusion criterion was older adult people with language barriers. The group conducted a one-week pre-survey before the formal survey to better modify the questionnaire. The survey mainly used face-to-face interviews to obtain data. A total of 3,530 older adult people were surveyed, 20 incomplete questionnaires were excluded, and 3,510 valid questionnaires were recovered.

### 2.2 Definitions and descriptions of variables

A structured questionnaire was developed to obtain the information. The dependent variable in this study was the anxiety score. The Generalized Anxiety Scale (GAD-7) was used to assess the anxiety status (29); this self-evaluation scale mainly uses the frequency of anxiety symptoms in the last 2 weeks as the evaluation criterion to determine the severity of anxiety. It has high reliability and validity (Cronbach’s coefficient: 0.91, cumulative variance contribution rate: 64.7%). The GAD-7 has a total of seven items with the total score ranging from 0 to 3 points; the response options for each item are divided into four categories, including never, a few days, more than half the time, and almost every day. A GAD-7 score ≥ 5 points indicates the presence of anxiety. The higher the score is, the more severe the respondents’ anxiety. The total anxiety score was calculated as the dependent variable.

The independent variable in this study was the activities of daily living (ADL) score. Adapted from the Lawton scale (30), eight questions as follows were used to assess ADL: (1) “Can you visit your neighbors by yourself?” (2) “Can you go shopping by yourself?” (3) “Can you cook a meal by yourself?” (4) “Can you wash clothing by yourself” (5) “Can you walk continuously for 1 kilometer at a time by yourself?” (6) “Can you lift heavy objects?” (7) “Can you continuously crouch and stand up three times?” (8) “Can you take public transportation by yourself?.” The response options are yes, with some difficulty, and no. The scores range from 1 to 3 points and the higher the score, the worse the respondents’ activities of living.

The mediating variable in this study was the social participation score. Social participation includes political participation, leisure participation, cultural inheritance, voluntary service, etc. (31). Due to the limitation of data, this paper pays attention to the leisure participation level of the older adult. According to the literature, the social leisure participation was measured from 11 main aspects (30). The participants were asked “Are you currently engaged in any of the following activities?,” including housework, tai chi, square dancing, socializing, planting flowers, reading books and newspapers, raising poultry (livestock), playing cards (mahjong), watching TV and listening to the radio, performing community organization activities, performing other outdoor activities, etc. The response options are (1) no; (2) occasionally; (3) at least once a month; (4) at least once a week; and (5) almost every day. Scores of 1 to 5 are given, with a higher score indicating greater social participation. The activities of daily living (ADL) and social participation was administered to participants as part of a home visit. A structured interview was subsequently conducted by a blinded interviewer.

The selection of control variables was mainly based on relevant literature, including gender (male or female), age (60–69 years old, 70–79 years old, 80 years old and above), marital status (married or single; single included divorced, widowed and unmarried participants), education level (primary school and below, junior school or high school, junior college and above), registered permanent residence (urban or rural), pension insurance status (no or yes), and health insurance status (no or yes) (see Table 1 for details).

**Table 1:** Control variables and assignments.

Variables	Variable assignment
Gender	Male = 1;Female = 0
Age	60–69 years old = 1;70–79 years old = 2;80 years old and above = 3
Marital status	Married = 1;Single = 0
Education level	Primary school and below = 1;Junior school or high school = 2;Junior college and above = 3
Registered permanent residence	Urban = 1; Rural = 0
Pension insurance status	Yes = 1;No = 0
Health insurance status	Yes = 1;No = 0

### 2.3 Procedure and statistical analysis

The study protocol was ethically reviewed and approved by the Ethics Committee of Xuzhou Medical University (XZHMU-2022085). Before the survey began, we explained the purpose of the study to the respondents. We also emphasized the confidentiality of the data, ensured the protection of their privacy, and obtained informed consent. Once the data were collected, we processed and analyzed them using SPSS software. The *t* test and variance analysis were used for univariate analysis. Multiple linear regression models were used for multivariate analysis. Pearson correlation analysis was used to study the correlation between the social participation levels and activities of daily living and anxiety. To explore the mediating role of social participation in the relationship between activities of daily living and anxiety, the Process program was used to conduct the bootstrap test, with the sampling number set at 5,000 and the confidence interval set at 95%. The significance level was set at *p* < 0.05.

## 3 Results

### 3.1 Older adult participants’ current levels of anxiety

The mean anxiety score of the surveyed older adult people was 3.028 ± 4.606 points. The participants’ current levels of anxiety are described in Table 2. According to the results in Table 2, among the 3,510 people surveyed, 1,006 had anxiety scores greater than or equal to 5 points (GAD-7 score ≥ 5), indicating that 28.66% of the older adult people had anxiety. See Table 2 for details.

**Table 2:** Anxiety scores of the older adult participants.

Anxiety score	Frequency	Percentage
GAD-7 score < 5	2,504	71.34
GAD-7 score ≥ 5	1,006	28.66
Sum	3,510	100.00

### 3.2 Univariate analysis of the older adult participants’ levels of anxiety

The results of the univariate analysis revealed statistically significant differences in the anxiety scores of the older adult participants stratified by sex, age, marital status, education status, and health insurance status (*p* < 0.05). Women had higher anxiety scores than men did. Respondents aged 60–69 years had higher anxiety scores. Single older adult people had higher anxiety scores than did married older adult people. Older adult people with a primary school education or less as well as those with a junior college education or above had higher anxiety scores. Older adult people with health insurance had lower anxiety scores than did those without health insurance. However, the anxiety scores of older adult people with different registered permanent residences and pension insurance statuses were not significantly different (see Table 3 for details).

**Table 3:** Results of univariate analysis of anxiety in older adult people.

Variable	Group	Anxiety score (mean value ± standard deviation)	*F/t*	*p*
Gender	Male	2.730 ± 4.350	3.409	0.001
	Female	3.263 ± 4.786		
Age	60–69 years old	3.484 ± 4.988	9.650	<0.001
	70–79 years old	2.757 ± 4.248		
	80 years old and above	2.787 ± 4.448		
Marital status	Married	2.422 ± 4.024	6.072	<0.001
	Single	3.374 ± 4.874		
Education level	Primary school and below	3.233 ± 4.777	14.780	<0.001
	Junior school or high school	2.191 ± 3.823		
	Junior college and above	3.270 ± 4.683		
Registered permanent residence	Urban	2.904 ± 4.659	1.106	0.269
	Rural	3.076 ± 4.500		
Pension insurance status	No	3.076 ± 4.639	0.922	0.357
	Yes	2.922 ± 4.352		
Health insurance status	No	3.319 ± 4.849	2.949	0.003
	Yes	2.836 ± 4.421		

### 3.3 Multifactorial analysis of anxiety in older adult people

A linear regression model including the anxiety score as the dependent variable and the activities of daily living score as the independent variable was developed. Model I showed that the anxiety score significantly increased by 0.172 points (*β*=0.172, *p* < 0.001) for every one-point increase in the activities of daily living score without including any control variables. Model 2 incorporated control variables based on Model 1. The results showed that the activities of daily living and anxiety scores of older adult people still exhibited a significant positive correlation (*β*=0.169, *p* < 0.001). For every one-point increase in the activities of daily living score, the anxiety score significantly increased by 0.169 points.

A linear regression model including the anxiety score as the dependent variable and the social participation score as the independent variable was developed. Model 3 showed that the anxiety score significantly decreased by 0.126 points (*β*= − 0.126, *p* < 0.001) for each one-point increase in the social participation score without including any control variables. Model 4 incorporated control variables based on Model 3. The results showed that the social participation score still exhibited a significant negative correlation with the anxiety score in older adult people (*β*= − 0.120, *p* < 0.001). For each one-point increase in the social participation score, the anxiety score significantly decreased by 0.120 points. Model 5 incorporated the activities of daily living score based on Model 4. The results showed that the effects of the social participation and activities of daily living score on the anxiety score in older adult people remained significant (*p* < 0.001). The activities of daily living score had a significant positive effect on the anxiety score in older adult people (*β*=0.122, *p* < 0.001), and the social participation score had a significant negative effect on the anxiety score in older adult people (*β* = − 0.058, *p* < 0.001) (see Table 4 for details).

**Table 4:** Results of the multifactorial analysis of anxiety in older adult people.

Variable (contrast group)	Anxiety				
	Model 1	Model 2	Model 3	Model 4	Model 5
Activities of daily living	0.172^***^ (0.011)	0.169^***^ (0.014)			0.122^***^ (0.018)
Social participation			−0.126^***^ (0.010)	−0.120^***^ (0.011)	−0.058^***^ (0.015)
Gender (female)		−0.106 (0.163)		−0.233 (0.165)	−0.168 (0.165)
Age (60–69)					
70–79 years old		0.165 (0.205)		−0.040 (0.203)	0.236 (0.206)
80 years old and above		−0.281 (0.186)		−0.440^*^ (0.187)	−0.240 (0.188)
Marital status (single)		0.009 (0.181)		−0.270 (0.179)	0.002 (0.183)
Education level (primary school and below)					
Junior school or high school		−0.384 (0.207)		−0.124 (0.216)	−0.155 (0.215)
Junior college and above		0.124 (0.242)		0.503^*^ (0.248)	0.334 (0.248)
Health insurance status (No)		−0.200 (0.163)		−0.154 (0.166)	−0.155 (0.165)
Constant item	0.490^***^ (0.184)	0.842^**^ (0.320)	5.647^***^ (0.220)	5.9963^***^ (0.252)	2.635^***^ (0.556)
*F*	219.22^***^	28.60^***^	170.84^***^	23.70^***^	26.39^***^
Adj-R^2^	0.0597	0.0626	0.0478	0.0528	0.0663

^***^p < 0.001, ^**^0.001 ≤ *p* < 0.01, ^*^0.01 ≤ *p* < 0.05.

### 3.4 Correlation analysis of social participation, activities of daily living, and anxiety in older adult people

Pearson’s correlation analysis revealed a significant negative correlation between the social participation and activities of daily living scores (*r* = −0.706, *p* < 0.001). There was a significant positive correlation between the activities of daily living and anxiety scores (*r* = 0.245, *p* < 0.001). There was a significant negative correlation between the social participation and anxiety scores (*r* = 0.219, *p* < 0.001) (see Table 5 for details).

**Table 5:** Correlation analysis of social participation, activities of daily living, and anxiety.

Variable	Activities of daily living	Social participation	Anxiety
Activities of daily living	1.000		
Social participation	−0.706^***^	1.000	
Anxiety	0.245^***^	−0.219^***^	1.000

^***^p < 0.001, ^**^0.001 ≤ *p* < 0.01, ^*^0.01 ≤ *p* < 0.05.

### 3.5 Analysis of the mediating effect of social participation in the relationship between activities of daily living and anxiety

According to Pearson’s correlation analysis, there was a two-by-two significant correlation among social participation, activities of daily living, and anxiety scores. To further validate the mediating effect of social participation in the relationship between activities of daily living and anxiety, this study used anxiety as the dependent variable (Y), activities of daily living as the independent variable (X), and social participation as the mediating variable (M). The Process program was also used to conduct bootstrap tests. The results showed that there was a significant positive relationship between the activities of daily living score and anxiety in older adult people (β =0.1719, *p* < 0.001), and the total effect was established. It also significantly affected the social participation score in older adult people (β = − 0.8560, *p* < 0.001). The direct effect of activities of daily living on anxiety in older adult people was significant after controlling for social participation (β =0.1225, *p* < 0.001). Social participation also had a significant negative effect on anxiety in older adult people (β = − 0.0542, *p* < 0.001). This finding indicated that the mediating effect of social participation in the relationship between activities of daily living and anxiety was established and partially mediated (see Table 6 for more details).

**Table 6:** Analysis of the mediating effect of social participation in the relationship between activities of daily living and anxiety.

Step	Regression equation		Fit index			Significance of the regression coefficient		
	Dependent variable	Independent variable	*R*	*R-sq*	*F*	β	*S.E.*	*t*
Step 1	Anxiety	Activities of daily living	0.246	0.061	215.620^***^	0.172	0.012	14.684^***^
Step 2	Social participation	Activities of daily living	0.706	0.499	3324.212^***^	−0.856	0.015	−57.656^***^
Step 3	Anxiety	Activities of daily living	0.255	0.065	116.229^***^	0.125	0.016	7.605^***^
		Social participation				−0.054	0.014	−3.985^***^

^***^p < 0.001.

**Figure 2:**
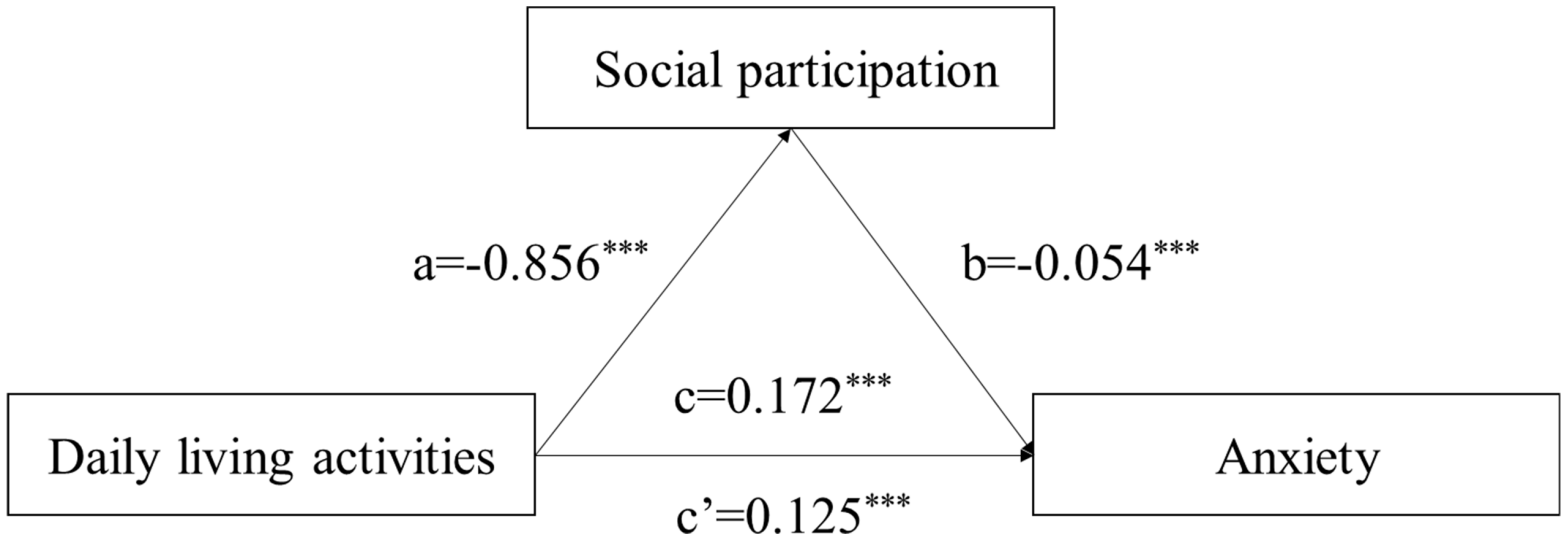
Modeling of the mediating effect of social participation in the relationship between activities of daily living and anxiety ^***^p < 0.001.

According to the results of the mediation effect analysis in Table 5, a standardized path model diagram was constructed (see Figure 2 for details).

The mediating effect of social participation was tested by the bootstrap method, and the results showed that the value of the direct effect was 0.1255, and the 95% confidence interval [0.0165, 0.0931] did not contain 0. Thus, a direct effect was established. The value of the indirect effect was 0.0464, and the 95% confidence interval [0.0272, 0.0662] also did not contain 0, indicating that an indirect effect was also established. Social participation played a significant mediating role in the relationship between activities of daily living and anxiety. According to the effect share calculation results, the indirect effect was 26.99% (see Table 7 for more details).

**Table 7:** Results of mediating effect analysis by bootstrapping.

Effect relationship	Effect value	BootSE	BootLLCI	BootULCI	Effect ratio
Total effect	0.172	0.012	0.149	0.195	
Direct effect	0.125	0.016	0.093	0.158	73.01
Indirect effect	0.046	0.010	0.027	0.066	26.99

BootLlCI refers to the lower limit of the 95% confidence interval, and BootULCI refers to the higher limit of the 95% confidence interval.

### 3.6 Robustness test

In order to ensure the robustness of the research results, the following two methods were adopted in this study to conduct the robustness test. The first approach is to transform the estimation model.1000 number of bootstrap samples was adopted. The results show that after transform the estimation model, the effect of ADL on anxiety in the older adult was still significant. The second approach is to incorporate new covariates, including household income, intergenerational support, and pension insurance, into the original regression model based on existing literature. After adding new covariates, the research results remain unchanged, indicating good robustness.

## 4 Discussion

Population aging is becoming increasingly serious. OECD report points out that population ageing is one reason why demand for healthcare and long-term care workers appears to be outstripping supply, with 18% of the population aged 65 and over on average in 2021 (32). Improving the health level of older adult people, maintaining their health function, and realizing “healthy aging” are major challenges faced by health workers. In recent years, scholars have begun to pay close attention to the health of older adult people, whose psychological health has increasingly become the core concern of all sectors of society (33). The physiological function of older adult people is decreasing, and the prevalence of chronic diseases is gradually increasing (34), which tends to cause more negative emotional reactions and serious psychological problems such as anxiety in older adult people (35, 36). Older adult people experience a decline in physical and psychological health, which in turn leads to lower life expectancy. Life expectancy at older ages bounced back from 2021 after a drop of about half a year in 2020 on average. However, since about 2012, the trend in life expectancy gains at age 65 has slowed down (37). It is highly important to improve the quality of life and happiness of older adult people by clarifying the factors influencing anxiety and improving their psychological health.

The mean anxiety score of the surveyed older adult people was 3.028 ± 4.606 points. Their current level of anxiety was determined. Among the 3,510 people surveyed, 1,006 had anxiety scores greater than or equal to 5 points (GAD-7 score ≥ 5), indicating that 28.66% of the older adult people had anxiety problems. Studies have shown that approximately 27.0% of older adult people in China have obvious psychological problems such as anxiety and depression (38). Therefore, the results of this study are not very different from those of previous studies. The results of the single factor analysis showed that the anxiety scores of women were greater than those of men. Previous studies also showed that female older adults reported higher levels of psychological distress (39). This may be because women under more stress than men because they have to divert some of their energy to family care after work. Moreover, women may be at greater risk of anxiety in old age due to chronic stress. Older people aged 60 to 69 years had higher anxiety scores. People in this age range generally face retirement, unemployment and significant declines in physical function and other problems. Changes in their social roles make it difficult for them to adapt quickly, so they are more prone to anxiety, depression and other negative emotions. Therefore, postponing the retirement age of the older adult can be considered. Research shows that increasing the retirement age of households has a large immediate improvement in household pension income (40). The increase in pension income will further improve the sense of security of the older adult, thereby reducing psychological anxiety. Therefore, China could consider making a policy to postpone the retirement age of the older adult. The results of this study also found that the anxiety levels of single older adult people were greater than those of married older adult people. People who do not have the companionship of a spouse may experience feelings of loneliness and alienation, especially those who have recently experienced the loss of a spouse, who may experience psychological trauma after facing this crisis event. The ensuing long-term loneliness increases the risk of anxiety in widowed older adult individuals. Older adult individuals with a primary school education or below and a college education or above have higher anxiety levels. Older adult people with a primary school education or below may not have social resources and receive social support in their later years. It is not easy for these individuals to solve difficult events, so they are more likely to be anxious. Older adult people with a junior college education or above may have greater expectations for their later life, so they are vulnerable to the impact of realistic pressure and are prone to anxiety. Older adult people with health insurance had lower levels of anxiety than did those without health insurance. The physical function of older adult people, who are prone to various physical and psychological diseases, declines in old age. Health insurance can make older adult people feel more secure and prevent poverty due to illness, thus reducing the risk of anxiety.

In addition to gender, age, marital status, education level and other characteristics affecting the anxiety levels of older adult people, activities of daily living also significantly affect their anxiety status. Activities of daily living are an important indicator for evaluating the health status of older adult people. To realize healthy aging is to reduce the time that older adult people cannot take care of themselves as much as possible in their later years. Most previous studies have focused on the relationship between impairment in activities of daily living and depression, and few studies have examined the relationship between impairment in activities of daily living and anxiety. However, there is a close relationship between activities of daily living and anxiety in older adult people. The results of this study showed that without the inclusion of any control variables, the anxiety score significantly increased by 0.172 points for every one-point increase in the activities of daily living score (β =0.172, *p* < 0.001). After the inclusion of control variables, the activities of daily living and anxiety scores of older adult people still showed a significant positive correlation (β =0.169, *p* < 0.001). For every one-point increase in the activities of daily living score, the anxiety score increased significantly by 0.169 points. The results show that activities of daily living are an important factor influencing anxiety in older adult people. The greater the activities of daily living score, the greater the anxiety score of older adult people. This finding is consistent with the results of previous studies. Studies have shown that impairment in activities of daily living reduces the range of activities of older adult people, their contact with the outside world and their opportunities to exchange information, making them prone to loneliness, anxiety, irritability and other psychological states, resulting in psychological problems (41). With impaired activities of daily living, older adult people are unable to assume corresponding social roles, thus disrupting their daily activities. As a result, they fail to control their negative psychology, which provides a suitable psychological environment for the generation of anxiety (42). The greater the level of activities of daily living, the lower the level of anxiety is. Improving the activities of daily living of older adult people can help reduce their anxiety levels (23).

To cope with aging, the WHO has developed a framework for active aging, which is mainly constructed from the aspects of “health, participation and security.” Among these aspects, “social participation” is the core component of active aging and a necessary way to realize active aging. At the same time, it is also an important factor influencing the psychological health of older adult people. The results of this study showed that for every one-point increase in the social participation score, the anxiety score significantly decreased by 0.126 points (β = − 0.126, *p* < 0.001) without the inclusion of any control variables. After the control variables were included, the social participation scores and anxiety levels of the older adult people still showed a significant negative correlation (β = − 0.120, *p* < 0.001). For every one-point increase in the social participation score, the anxiety score significantly decreased by 0.120 points. This shows that social participation is also an important factor influencing anxiety in older adult people. Studies have shown that social participation can improve the psychological health of older adult people (43) and has a significant impact. With the increase in social participation types, their psychological state improves (44). Some scholars have also found that social participation affects activities of daily living and depression in older adult people. The greater the social participation score was, the greater the activities of daily living score and the less severe the depression (45). In summary, previous studies have begun to pay attention to the relationship between social participation and the psychological health of older adult people. However, we found that scholars have mostly studied the impact of social participation on depression and psychological health, and few have focused on the impact of social participation on anxiety. Through analysis, this study revealed that social participation is also an important factor influencing anxiety in older adult people. Strengthening the social participation of the older adult can develop the human resources, effectively reflect the personal value of the older adult, reduce the anxiety of the older adult, and improve the psychological health. Nowadays, the use of digital technology is becoming more and more common, and digital intelligence literacy has a positive effect on the social participation of the older adult. It is suggested to strengthen the aging adaptation and anti-addiction construction, broaden and simplify the access to digital technology services, so as to improve the level of social participation of the older adult. In addition, community organization culture and entertainment culture also have a positive impact on the social participation of the older adult. Community institutions should strengthen cultural construction and improve the level of social participation of the older adult.

In summary, activities of daily living and social participation have a significant impact on anxiety in older adult people. There is a complex relationship among the three variables. Studying the relationships among these variables will help to better reduce the anxiety levels of older adult people and improve their psychological health and quality of life. Through Pearson correlation analysis, we found that activities of daily living were negatively correlated with social participation (*r* = −0.706, *p* < 0.001) but positively correlated with anxiety (*r* = 0.245, *p* < 0.001). There was a significant negative correlation between social participation and anxiety in older adult people (*r* = −0.219, *p* < 0.001). This provides a theoretical basis for further verification of the mediating effect of social participation in the relationship between activities of daily living and anxiety. In this study, anxiety was taken as the dependent variable (Y), activities of daily living was taken as the independent variable (X), and social participation was taken as the mediating variable (M). A bootstrap test was conducted using the Process program. The results showed that activities of daily living had a significant positive effect on anxiety in older adult people (β =0.1719, *p* < 0.001), and an overall effect was established. Activities of daily living also significantly affected the social participation level of older adult people (β = − 0.8560, *p* < 0.001). After controlling for social participation, the direct effect of activities of daily living on anxiety in older adult people was still significant (β =0.1255, *p* < 0.001). Social participation had a significant negative effect on anxiety in older adult people (β = − 0.0542, *p* < 0.001). The results of the bootstrap test showed a partial mediating effect of social participation in the relationship between activities of daily living and anxiety. This indicates that the activities of daily living of older adult people can not only directly affect their anxiety levels but also have an impact on their anxiety through their social participation. Social participation played an obvious mediating role in the relationship between activities of daily living and anxiety. The greater the activities of daily living score was, the greater the respondents’ levels of social participation. Decreased activities of daily living limit the social activities of older adult people and affect their social participation. Older people with greater social participation can exercise their living abilities through various social activities, such as socializing, shopping, and participating in community organization activities, to reduce the risk of illness and experience positive impacts on their psychological health.

## 5 Conclusion

The results of this study revealed that 28.66% of the older adult participants had anxiety. Activities of daily living and social participation are important factors influencing anxiety. Social participation played a mediating role in the relationship between activities of daily living and anxiety. This shows that improving the activities of daily living and social participation of older adult people is very important for reducing their anxiety levels and improving their psychological health. Therefore, more health education should be carried out to enhance physical exercise. The proper intensity and a reasonable form of physical exercise can strengthen the physiological function of each organ system and improve activities of daily living in older adult people. In addition, efforts should be made to create a good atmosphere for promoting social participation among older adult people, thus improving their subjective initiative and the sustainability of social participation. Moreover, community-based social activity platforms for older adult people should be actively constructed to improve their psychological health. Community institutions can carry out various forms of cultural education and training, as well as various forms of cultural and recreational activities, such as chess and card activities, square dancing, to encourage more older adult people to participate in social activities. In addition, with the development of digital intelligence, digital intelligence literacy has a positive effect on the social participation of the older adult. Community institutions should strengthen the “science and technology care” for the older adult to adapt to the digital age, develop targeted digital products and digital experience environment areas to improve the level of social participation of the older adult.

The innovation of this research is mainly reflected in the following two aspects: First of all, although there is a lot of research on the impact of social participation on mental health in older adults, little research has been reported on the relationship between daily living ability, social participation, and anxiety in the older adult people. We hypothesized that the anxiety is the dependent variable, ADL is the independent variable, and social participation is the mediating variable, so as to explore the influencing mechanisms of the effects of activities of daily living on anxiety in older adult people. Secondly, this study comprehensively explored the relationship between daily activity ability and anxiety in the older adult. It explains the potential factors between daily activity ability and anxiety, and makes clear that the association between daily activity ability and anxiety is realized through the mediating role of social participation. These findings provide useful theoretical and practical significance for the early detection and prevention of anxiety in the older adult.

## Data Availability

The datasets presented in this article are not readily available because the data that support the findings of this study are available on request from the corresponding author. The data are not publicly available due to privacy or ethical restrictions. Requests to access the datasets should be directed to alicezj2018@xzhmu.edu.cn.
